# Endovascular therapy and minimally invasive coronary artery bypass grafting for Leriche syndrome with ischaemic heart disease

**DOI:** 10.1093/icvts/ivaf008

**Published:** 2025-01-22

**Authors:** Hiroki Moriuchi, Mamoru Orii, Nobuhiro Shimabukuro, Akihiko Yamauchi

**Affiliations:** Department of Cardiovascular Surgery, Yuuai Medical Center, Okinawa, Japan; Department of Cardiovascular Surgery, Yuuai Medical Center, Okinawa, Japan; Department of Cardiovascular Surgery, Yuuai Medical Center, Okinawa, Japan; Department of Cardiovascular Surgery, Yuuai Medical Center, Okinawa, Japan

**Keywords:** Leriche syndrome, endovascular therapy, minimally invasive coronary artery bypass grafting

## Abstract

In patients with Leriche syndrome and coronary artery disease, the operative strategy is very important because the internal thoracic artery often provides important collateral blood flow to the lower extremities. A 65-year-old man with diabetes mellitus was admitted with heart failure and bilateral claudication. We successfully performed endovascular therapy for aortoiliac occlusive disease, followed by minimally invasive coronary artery bypass grafting for ischaemic heart disease. Postoperative course was uneventful. This is the first report of using minimally invasive coronary artery bypass grafting and endovascular therapy to treat Leriche syndrome with ischaemic heart disease.

## INTRODUCTION

In patients with Leriche syndrome, the internal thoracic artery (ITA) often provides important collateral blood flow to the lower extremities. There is concern about lower limb ischaemia when the ITA is used as a graft in coronary artery bypass grafting (CABG). We report the first case of a patient with Leriche syndrome and ischaemic heart disease (IHD) treated with minimally invasive coronary artery bypass grafting (MICS-CABG), preceded by endovascular therapy (EVT). Written informed consent was obtained from the patient.

## CASE REPORT

**Figure 1:**
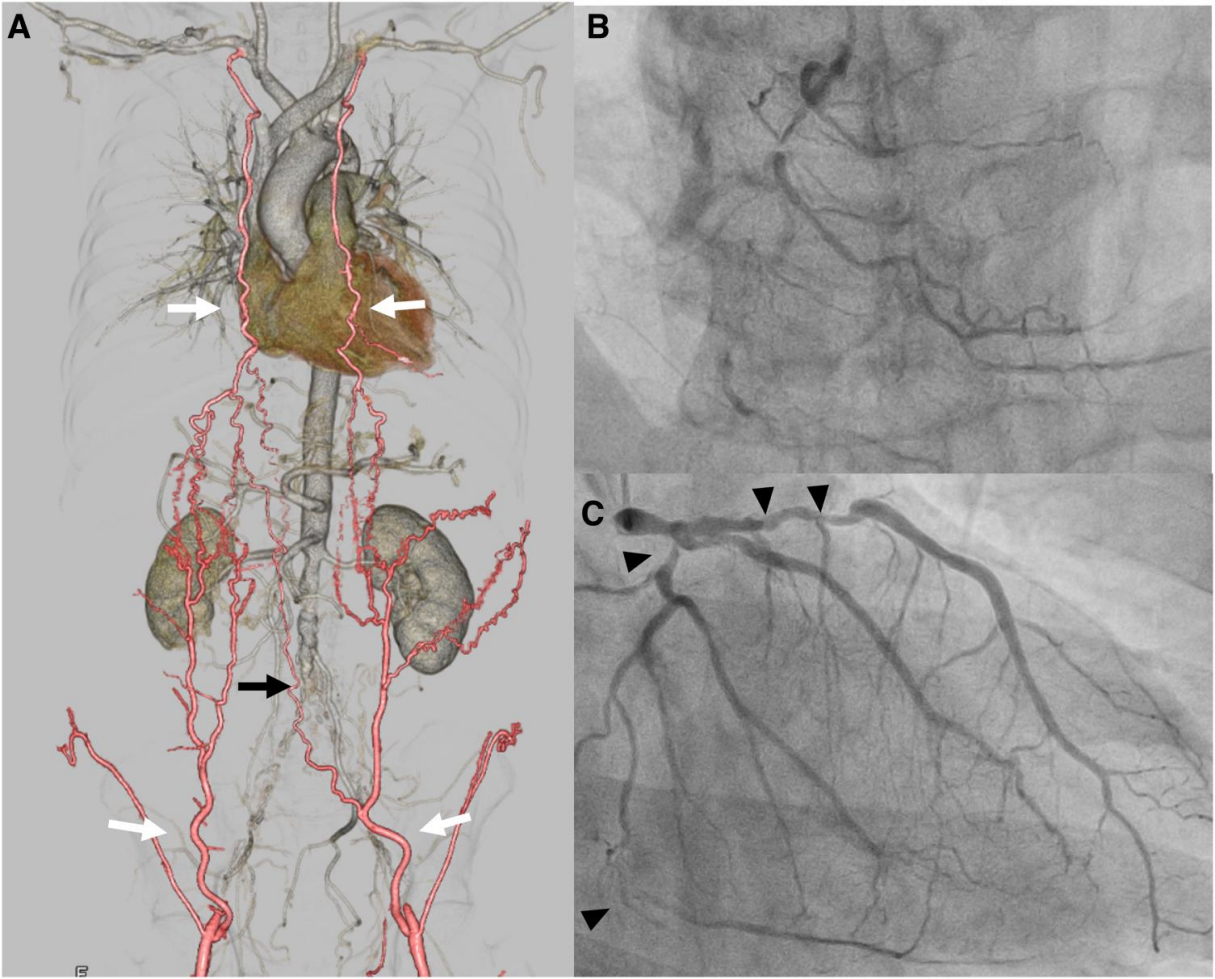
(**A**) 3D computed tomography showed the occlusion of infrarenal aorta and bilateral common iliac artery (black arrow). Inferior epigastric artery from ITA provided collateral flow to the lower extremities (white arrow). (**B** and **C**) Coronary angiography showed total occlusion of RCA and severe stenosis of LAD and LCX (arrow head). ITA: internal thoracic artery; LAD: left anterior descending artery; LCX: left circumflex artery; RCA: right coronary artery

A 65-year-old man with diabetes mellitus (DM) was admitted with heart failure and bilateral claudication. Echocardiography showed left ventricular ejection fraction of 47%, and coronary angiography revealed severe triple coronary artery disease (Fig. 1).

Contrast-enhanced computed tomography showed total occlusion of the infra-renal abdominal aorta and bilateral common iliac arteries, consistent with Leriche syndrome. Blood flow to the lower extremities was supplied by the inferior epigastric artery originating from the ITA (Fig. 1). To achieve a minimally invasive approach and enhance long-term prognosis, we decided to perform EVT, followed by MICS-CABG.

A 8 Fr sheath (TERUMO, Tokyo, Japan) was inserted into the common femoral artery on both sides, and a 6 Fr guiding sheath Crossroad (Nipro, Osaka, Japan) was placed in the left brachial artery. After heparinization, aortography revealed the complete occlusion of the infrarenal abdominal aorta. Using bidirectional approach via the brachial artery and femoral artery, we successfully crossed the occlusion site with 5 Fr Tempo 125 cm (Cordis, Cardinal Health, OH, USA), 1.5 Fr ASAHI Corsair Armet 150 cm (ASAHI INTECC Co Ltd, Aichi, Japan) and 0.014 guidewire ASAHI Gladius MG14 PV 235 cm (ASAHI). After predilation with 3 × 40 mm JADE balloon (OrbusNeich, Hong Kong), the 0.014 wire was replaced with a 0.035 radifocus wire (TERUMO) in both femoral arteries. A 14 × 60 mm SMART stent (Cordis) was deployed 1 cm peripherally from the renal artery bifurcation. Subsequently, two 7 × 79 mm VBX stents (W. L. Gore, Flagstaff, AZ, USA) and one 10 × 60 mm SMART stent were inserted from the right femoral artery, followed by two 8 × 79 mm VBX stents and one 10 × 60 mm SMART stent inserted from the left femoral artery. We deployed them and dilated using a 8 × 100 mm SHIDEN HP (Kaneka Medix, Osaka, Japan) simultaneously on both sides.

**Figure 2:**
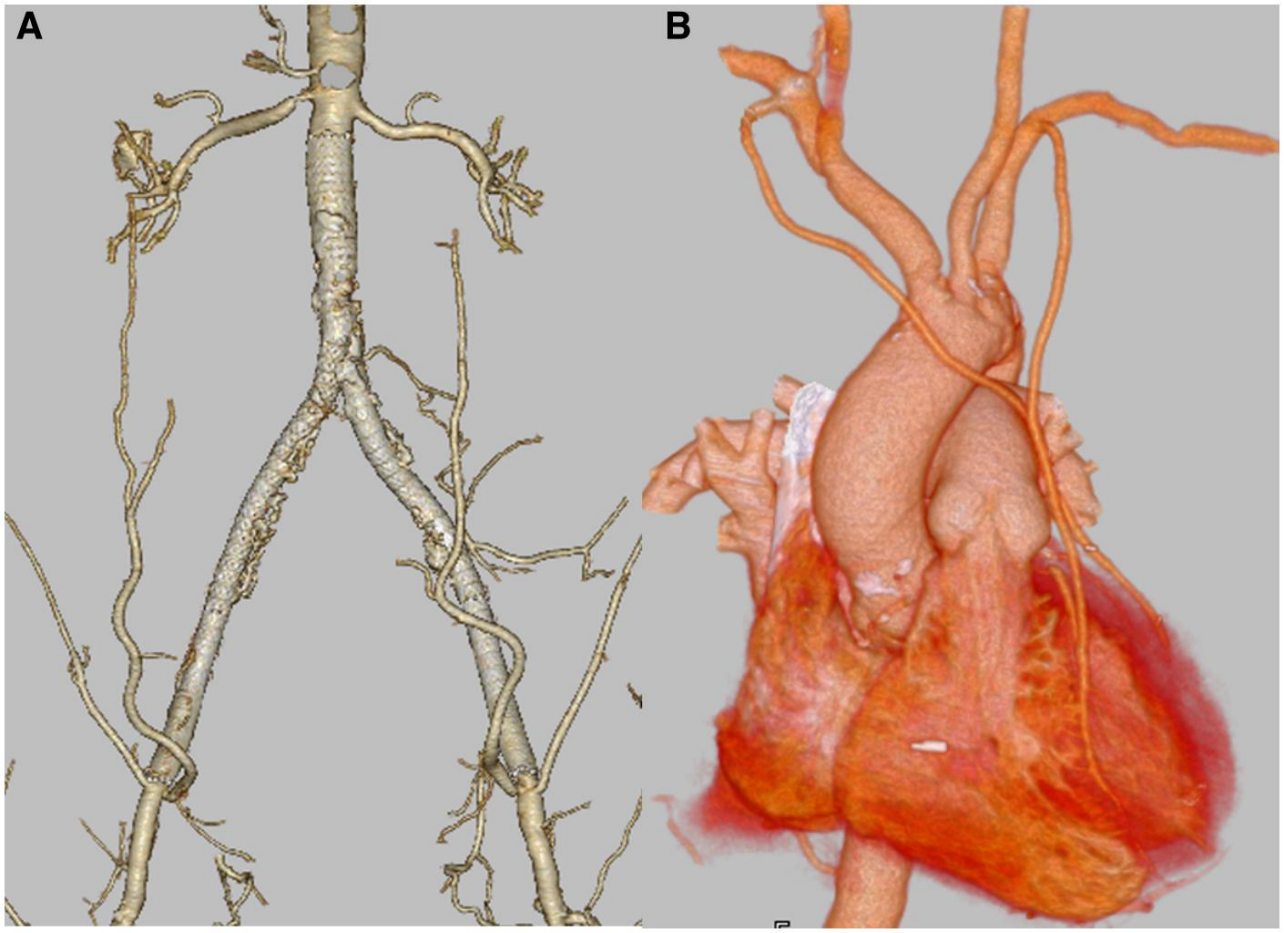
(**A** and **B**) Postoperative 3D computed tomography showed good blood flow of the aortoiliac artery and patent bypass

Post EVT course was good, we performed MICS-CABG 2 weeks after EVT. Under general anaesthesia with differential lung ventilation, the patient was positioned in the right lateral decubitus position. A 9-cm thoracotomy was performed in the left fifth intercostal space, and a port was inserted dorsal to the incision. The Thoratrak retractor (Medtronic, Inc., MN, USA) was used to open the ribs and retract them. Bilateral ITAs were harvested in a skeletonized fashion using a 32-cm Harmonic scalpel (Ethicon Endo-Surgery, Inc., USA). The right anterior second intercostal space was opened, and the right ITA was guided out of the chest wall. An I-composite graft was created by anastomosing the right ITA with the radial artery harvested from the right arm. The heart was lifted using three deep pericardial sutures, securing the field of vision. The I-composite graft was anastomosed to the posterolateral artery of the left circumflex artery and the posterior descending artery of the right coronary artery; the left ITA was grafted to the left anterior descending artery. All anastomosis was performed under off pump beating with epicardial stabilizer (Medtronic). The operation was completed after confirming good graft flow. Clopidogrel 75 mg/day was initiated on the first postoperative day. Postoperative course was uneventful, and postoperative computed tomography showed good blood flow to the lower extremities and patent graft (Fig. 2).

## DISCUSSION

Leriche syndrome is associated with a high incidence of IHD, and the surgical strategy is very important because the use of the ITA as a graft for CABG may worsen lower limb ischaemia [1].

Surgical bypass has been used for lower extremities revascularization, EVT in the aorto-iliac artery region has a primary patency rate of 91.9% and a secondary patency rate of 98.5% at 5 years [2].

CABG is reported to have a better prognosis than percutaneous coronary intervention in patients with multivessel disease and DM. The use of bilateral ITAs in patients with DM improves survival and decreases coronary reintervention compared to single ITA [3]. MICS-CABG is less invasive than conventional CABG and reduces the risk of mediastinitis.

In this case, we performed EVT first to prevent lower limb ischaemia following CABG. Off-pump MICS-CABG with total arterial graft was chosen to improve long-term prognosis with minimal invasiveness. We believe this strategy is the best in this case; however, long-term follow-up is necessary.

## CONCLUSION

This is the first report of using MICS-CABG and EVT to treat Leriche syndrome with IHD. In patients with Leriche syndrome and IHD, the severity and anatomy vary from case to case. Therefore, careful preoperative planning is essential.

## Data Availability

All relevant data are within the manuscript.
